# Biological features of fowl adenovirus serotype-4

**DOI:** 10.3389/fcimb.2024.1370414

**Published:** 2024-06-10

**Authors:** Farooq Rashid, Zhixun Xie, You Wei, Zhiqin Xie, Liji Xie, Meng Li, Sisi Luo

**Affiliations:** ^1^ Department of Biotechnology, Guangxi Veterinary Research Institute, Nanning, China; ^2^ Guangxi Key Laboratory of Veterinary Biotechnology, Nanning, China; ^3^ Key Laboratory of China (Guangxi)-ASEAN Cross-border Animal Disease Prevention and Control, Ministry of Agriculture and Rural Affairs of China, Nanning, China

**Keywords:** FAdV-4, epidemiology, coinfection, structural proteins, mutations

## Abstract

Fowl adenovirus serotype 4 (FAdV-4) is highly pathogenic to broilers aged 3 to 5 weeks and has caused considerable economic loss in the poultry industry worldwide. FAdV-4 is the causative agent of hydropericardium-hepatitis syndrome (HHS) or hydropericardium syndrome (HPS). The virus targets mainly the liver, and HPS symptoms are observed in infected chickens. This disease was first reported in Pakistan but has now spread worldwide, and over time, various deletions in the FAdV genome and mutations in its major structural proteins have been detected. This review provides detailed information about FAdV-4 genome organization, physiological features, epidemiology, coinfection with other viruses, and host immune suppression. Moreover, we investigated the role and functions of important structural proteins in FAdV-4 pathogenesis. Finally, the potential regulatory effects of FAdV-4 infection on ncRNAs are also discussed.

## Introduction

Fowl adenoviruses (FAdVs) are the causative agents of many clinical diseases in poultry and have caused substantial economic loss to the poultry industry worldwide (Schachner et al., 2018). FAdVs are classified under the family *Adenoviridae* and genus *Aviadenovirus* and are further divided into five species, from A to E, based on their restriction enzyme digestion profile (Zsák and Kisary, 1984). The International Committee on Taxonomy of Viruses, based on cross-neutralization tests results, has divided FAdVs into 12 serotypes (Hess, 2000; Balamurugan and Kataria, 2004; Kim et al., 2014). The typical diseases in chickens caused by adenoviruses include hepatitis-hydropericardium syndrome (HHS), which is caused by FAdV-4 (species FAdV-C) (Mettifogo et al., 2014; Li H. et al., 2016); inclusion body hepatitis (IBH), which is caused by FAdV-8 and -11 (species FAdV-E and FAdV-D, respectively) (Nakamura et al., 2011; Schachner et al., 2016); and adenoviral gizzard erosion (GE), which is caused by FAdV-1 infections (belonging to FAdV-A) (Domanska-Blicharz et al., 2011; Thanasut et al., 2017). It has been reported that FAdVs are distributed worldwide, and different serotypes have been found in different geographical locations and subsequently spread to various regions (Alemnesh et al., 2012; Mase et al., 2020).

FAdV-4 was first reported in the Angara region of Pakistan in 1987. This disease subsequently spread to other regions of the world and caused enormous economic losses in the poultry sector (Afzal et al., 1991). The virus is nonenveloped, and its genome is double-stranded DNA of approximately 45 kb. HHS or hydropericardium syndrome (HPS) mainly affects chickens aged 3- to 6-weeks (Balamurugan and Kataria, 2004). Ruffled feathers, lethargy, prostration and asitia are common clinical symptoms. The accumulation of clear, straw-colored fluid in the pericardial sac is a typical sign of a necrotic lesion. The liver also shows multifocal areas of necrosis and hepatitis (Nakamura, 1999). Moreover, HPS has also been observed in other birds, such as pigeons, quails, ducks and mandarin ducks (Hess et al., 1998; Roy et al., 2004; Pan et al., 2017; Shen et al., 2019).

In China, FAdV-4 outbreaks were reported earlier (Li H. et al., 2016), but since June 2015, dramatic increases in the incidence of FAdV-4 outbreaks have been reported in several provinces, including Anhui, Henan, Hubei, Jiangsu, Jiangxi, Shandong, and Zhejiang (Li et al., 2017), with higher mortality rates in 3–5-week-old broiler chickens than in previous HPS outbreaks caused by FAdVs. Infection with these hypervirulent isolates of FAdV-4 resulted in 80%–100% mortality (Ye et al., 2016). The Chinese FAdV-4 isolates share about 99.9% -100% genetic identities among themselves, and isolates from other countries (KR5, ON1, MXSHP95, and B1-7) also share nucleotide identities ranging from 97.7% - 98.9% among themselves. However, lower identities were obsrved between isolates from the other countries (KR5, ON1, MXSHP95, and B1-7) and Chinese isolates. In the current review, we discuss the different aspects of FAdV-4 infection, e.g., epidemiology, physiological features, coinfections with other viruses, comparisons of nonpathogenic and highly pathogenic FAdV-4 strains, major proteins and their functions, and effects on host immune responses. Moreover, we have discussed the regulatory effect of FAdV-4 infection on noncoding RNAs (ncRNAs).

## Epidemiology of FAdV-4

Studies have confirmed that broiler chickens are the most affected targets of FAdV-4 infection. When chickens are infected with FAdV-4, they most likely will develop HPS (Grgić et al., 2013a). The first outbreak of FAdV-4 was reported in Pakistan in Angara Goth, near Karachi, in 1987, in which the mortality rate was high in broiler chickens aged 3–5 weeks (Khawaja et al., 1988; Grgić et al., 2013a). In India, HHS was known as “lychee” disease, and subsequently, the disease spread to other geographical regions, including India (Gowda and Satyanarayana, 1994), Iraq (al-Attar, 1991), Japan (Mase et al., 2012), Central and South America (Cowen et al., 1996; Shane, 1996; Toro et al., 1999), Russia (Borisov et al., 1997), Slovakia (Jantosovic et al., 1991), Bangladesh (Biswas et al., 2002), Korea (Choi et al., 2012), China (Li L. et al., 2016; Li et al., 2017) Poland (Niczyporuk, 2016), Iran (Mirzazadeh et al., 2020), Brazil (De la Torre et al., 2018), Croatia (Zadravec et al., 2011), Australia (Steer et al., 2011), Greece (Franzo et al., 2020), the United Arab Emirates (Ishag et al., 2022), Saudi Arabia (Hemida and Alhammadi, 2017), Morocco (Abghour et al., 2019), and South Africa (Venter, 2014), with a mortality rate of 20–80%. FAdV-4 infections are mostly endemic during humid and hot weather (Schachner et al., 2014; Shah et al., 2016); however, during other weather conditions, sporadic outbreaks can also occur. In China, surveillance of FAdV-4 during 2006 and 2014 revealed sporadic or clustered distributions of the virus (Li L. et al., 2016), but since July 2015, epidemics have been observed in several provinces of China, such as Anhui, Xinjiang, Shandong, Liaoning Hebei, Jilin, Heilongjiang, Jiangxi, Henan, Jiangsu, and Hubei, where the virus tends to spread rapidly (Shane, 1996; Li L. et al., 2016). The mortality in these regions was approximately 40%, reaching 90% in serious cases and causing substantial economic loss. Phylogenetic analysis of the hexon gene revealed that a FAdV-4 outbreak was associated with two Chinese isolates (PK-01 and PK-06) that resided on a comparatively independent branch of the tree. This analysis revealed that Chinese strains might have originated from Indian strains (Changjing et al., 2016; Zhang et al., 2016).

The FAdV-4 Chinese isolates that have a truncated ORF19 gene are considered responsible for this pandemic (Li L. et al., 2016; Ye et al., 2016). However, further research is needed to determine the mechanism of ORF19 deletion in highly pathogenic strains. Furthermore, compared with those of a 2013 Chinese isolate, the ORF29 genes of these Chinese isolates contained 33-nt or 66-nt deletions (Ye et al., 2016), suggesting that these FAdV-4 strains have adapted to their hosts. Similarly, FAdV-4 detected in ducks, geese and ostriches caused the same symptoms as HPS in chickens (Ivanics et al., 2010; Li L. et al., 2016; Chen et al., 2017). In ducks aged 25–40 days, the mortality rate is 15–30% (Chen et al., 2017). In goslings, morbidity and mortality begin at 8–9 days and continue until 24–25 days of age (Toro et al., 1999). In ostriches, the mortality rate is 15–30%, and death occurs at a young age (Shane, 1996). These epidemiological data are important because they are helpful in determining the relationships of this virus in different host species and thus can help in the control of HPS.

## Physiological features of FAdV-4

Various reports have shown that FAdV-4 cells are approximately 80 to 90 nanometers (nm) in diameter and filterable through a 0.1 μm pore size membrane. Transmission electron microscopy (TEM) of the virus from liver extract indicated that the virus was isometric and spherical (Chandra et al., 2000). The virus can be inactivated by heating at 60°C for 1 h, 80°C for 10 min, or 100°C for 5 min or by treatment with 5% chloroform or 10% ether. In addition, it can tolerate pH values ranging from 3 to 10 (Afzal et al., 1991). Under natural conditions, wild birds are carriers of FAdV-4 (Manzoor et al., 2013), which can also be transmitted to healthy birds through subcutaneous or intramuscular injection of virus-infected liver homogenate from infected birds (Naeem et al., 2001). The highly pathogenic FAdV-4 serotype of broilers is naturally transmitted both vertically and horizontally (Asthana et al., 2013). In the former case, FAdV-4 infection of broiler chickens prior to egg laying was most likely congenital because the virus survives in the eggs (Tahir et al., 2017). In the latter case, the virus from the infected chicken is transferred to healthy flocks through the fecal-oral route because of the high shedding titer in feces (Chandra et al., 2000); however, airborne and aerosol transmission of FAdV-4 is negligible. The severity of the pathogenicity of FAdV-4 infection is reportedly related to the mode of infection (Mazaheri et al., 1998). Infection with HN/151025 in 7-, 21-, and 35-d-old chickens led to 90%, 30%, and 28.6% mortality, respectively, when administered intranasally or intramuscularly. These results indicate that the intramuscular route is the most sensitive for detecting FAdV-4 infection in chickens (Mazaheri et al., 1998; Changjing et al., 2016; Liu et al., 2016). HHS is characterized by depression, decreased feed intake, neck feather ruffling, and lethargy (Liu et al., 2016).

The common gross lesions of HPS in chickens include the accumulation of clear or yellowish jelly-like fluid in the pericardial sac and hydropericardium and a yellow−brown-brown color and swollen, congested, enlarged, and friable with foci of necrosis and hemorrhages in the liver. In some cases, hemorrhages occur in the kidneys, spleens and lungs (Sun et al., 2019). Additionally, an enlarged bursa of Fabricius has also been observed (Wu et al., 2020). FAdV-4 target the chicken hepatic cells and therefore this virus is highly pathogenic to chickens (Chen et al., 2019). The incubation period of FAdV-4 disease has been reported to be 24 to 48 hours after infection trough natural routes (Ren et al., 2019).

The genome of nonpathogenic FAdV-4 consists of 23.3% A, 27.7% C, 26.9% G and 22.1% T, with a G+C content of 54.6% (Griffin and Nagy, 2011). The FAdV genome encodes 10 structural proteins, namely, hexon, penton base, fiber-1, fiber-2, terminal protein (TP); and proteins VI, VII, VIII, III, and μ (Li et al., 2017), and 11 nonstructural proteins. The major structural proteins include Hexon, which forms the facets of the icosahedral structure; the penton base, which covers the vertices of the capsid; and two antenna-like fiber proteins (Fiber-1 and Fiber-2) anchored in each penton base protein (Hess et al., 1995). Similarly, the four core structural proteins include Tp, μ, pV, and pVII, which form a complex with the genomic DNA inside the viral particles (Nemerow et al., 2009). Similarly, three minor capsid proteins, protein IIIa (pIIIa), protein VI (pVI), and protein VIII (pVIII), are embedded in the inner surface of the capsid structure and play major roles in virion structural integrity (Zhao et al., 2015). The nonstructural proteins include DNA-binding Protein (DBP; also known as E2A), E1A, E1B, ADP (also known as E3), E4, EP, pol, pIVaII, 33 K, 100 K and 52/55 K (Hess et al., 1995; Nemerow et al., 2009; Griffin and Nagy, 2011; Xie et al., 2013).

**Figure 1:**
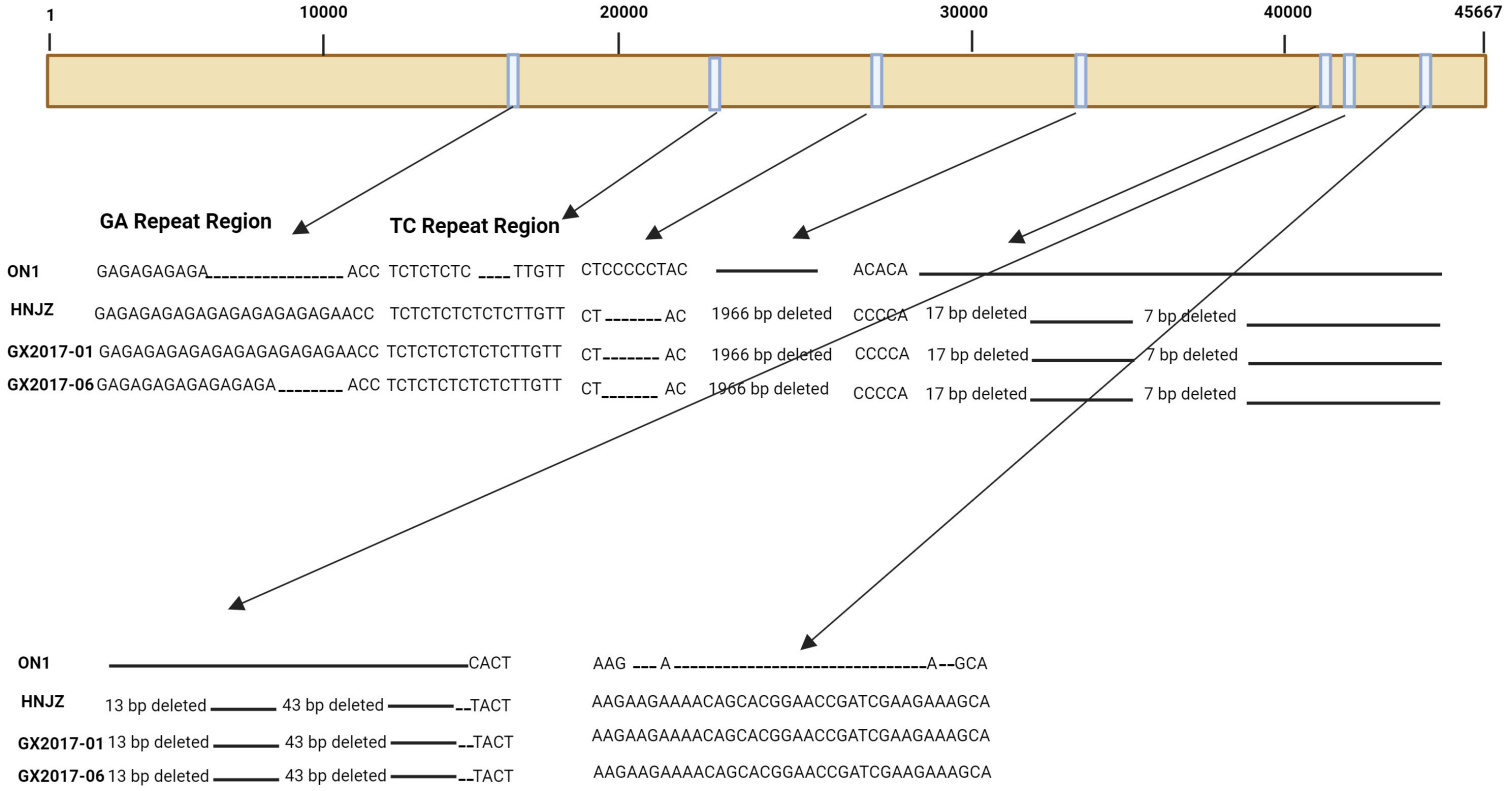
Schematic representation of the genome of three FAdV-4 isolates representing major insertions and deletions at various sites when compared to ON1 strain of FAdV-4.

Compared with non-Chinese isolates, FAdV-4 isolates from China have been reported to have significant deletions and mutations in their genome (Liu et al., 2016; Ye et al., 2016). All the pathogenic strains isolated in China have 10-bp and 3-bp insertions in tandem repeat region B (TR-B) and a 55 kDa protein-coding sequence, respectively, while a 6-bp deletion was detected in the 33 kDa protein-coding fragment. Moreover, a 1966-bp deletion has been observed in isolates from China (**Figure 1**), resulting in the removal of open reading frame (ORF) 19, ORF27, and ORF48. All the isolates from China also have longer GA repeats between the pX and pVI genes compared to the isolates from other countries (**Figure 1**). In addition, amino acid mutations were also observed in structural genes of Chinese isolates compared to those in isolates from other countries.

## Coinfection of FAdV-4 with other serotypes and avian viruses

In the poultry industry, coinfections of FAdV with other pathogens have been reported regularly. Several reports have indicated that coinfections of FAdV-4 with different serotypes of FAdVs as well as with other immunosuppressive pathogens produce various synergistic effects, as exemplified by coinfections of FAdV-4 with the *infectious bursal disease virus* (IBDV), which induces immunosuppression and enhances the pathogenicity of FAdV-4 (Xu et al., 2021). Similarly, coinfection of chickens with FAdV-4 and *chicken infectious anemia virus* (CIAV) and *Avibacterium paragallinarum* resulted in significantly increased mortality compared to with infection caused by FAdV-4 alone (Mei et al., 2020). However, coinfection of FAdV-4 with serotype FAdV-8a in specific-pathogen-free (SPF) chickens resulted in decreased proliferation and replication of FAdV-4 while enhancing the proliferation and replication of FAdV-8a in chickens (Liu et al., 2021). A recent study compared coinfection with FAdV-4 and duck circovirus (DuCV) and monoinfection (Shen et al., 2023). DuCV is a prevalent infectious virus in the duck industry in China. The incidence of FAdV-4 infection in ducks has increased in recent years. A systematic study of Cherry Valley ducks revealed more serious clinical signs and symptoms in those coinfected with FAdV-4 and DuCV than in those with a monoinfection. These pronounced signs included pericardial effusion, immunosuppression, and hepatitis. Moreover, the different organs showed greater viral loads, immune indices and biochemical indices in these ducks than in those with a monoinfection. According to further experimental findings, the coinfected ducks exhibited significantly greater viral replication and more tissue damage than did the monoinfected ducks. The data obtained from coinfection studies indicate that strict monitoring and preventive measures need to be taken in the field to control the severity of coinfections caused by FAdV-4 and other infectious agents.

## Comparison of nonpathogenic and highly pathogenic FAdV-4 isolates

When comparing nonpathogenic strains of FAdV-4 with pathogenic strains, a large difference has been observed. A typical example of a nonpathogenic strain of FAdV-4 is ON1, which was isolated in 2004 from a broiler breeder flock in Canada that showed no clinical signs of HPS (Griffin and Nagy, 2011). The genome size of ON1 is 45667 bp, containing 23.3%, 27.7%, 26.9% and 22.1% A, C, G, and T, respectively, with a G+C content of 54.6%. This strain contains 46 protein-coding ORFs. To evaluate the pathogenic potential of ON1, a trial was conducted on chickens, and the virus was administered either orally or intramuscularly. The usual signs of HHS, such as huddling of chickens in corners with ruffled feathers, depression, or a specific posture of chickens with their chest and beak resting on the ground, were not observed. Furthermore, the dissected chickens also did not exhibit hydropericardium or any gross lesions. The liver seemed normal, and no inclusion bodies were detected in hepatocytes.

Severe FAdV-4 infection cases with IBH and HPS have been observed in China since 2013 (Zhao et al., 2015), and since then, highly pathogenic FAdV-4 strains have been found throughout China. There was a greater number of changes in the highly pathogenic isolates from China during different years than in the ON1 strain from Canada. The major changes observed were as follows: a 6-bp deletion at positions 28,784 to 28,789 and a 1,966 bp deletion at the 3’ end of the genome at positions 35,389 to 37,354. The 1,966-bp deletion has been reported in all FAdV-4 isolates from China thus far (**Figure 1**) (Ganesh et al., 2001; Roberts et al., 2006; Matsushima et al., 2011); a 43-bp deletion at positions 41,766 to 41,809 was also observed (**Figure 1**). Furthermore, other deletions at 41,675 bp to 41,691 bp, 41,704 bp to 41,710 bp, and 41,748 bp to 41,760 bp have also been observed. In addition to deletions, insertions were also observed in these isolates compared to those in the ON1 strain. A major 27-bp insertion in ORF19 A at position 44,376 was also observed. Since all the highly pathogenic FAdV-4 strains from China had the deletion of 1,966 bp, this deletion was explored for its role in pathogenicity; however, it was determined to be dispensable for the development of highly pathogenic FAdV-4 isolates (Pan et al., 2017). The naturally occurring mutations are rare in DNA viruses as their genomes are considered very stable. However, besides FAdV-4, another DNA virus; hepatitis B virus (HBV) was also found to have naturally occurring mutations (Peng et al., 2015). The transcription of HBV genome is under the control of four promoters i.e. X, S, preS1, and C. Among these, the core promoter (CP) transcribe the precore (preC) and pregenomic RNA (pgRNA) mRNA, and thus the CP play significant role in viral replication (Quarleri, 2014).Point mutations in CP greatly affect virus expression and replication, and therefore disease development and progression are directly related to mutations in CP (Peng et al., 2015). Since, in FAdV-4 also the mutations are natural and therefore must be thoroughly investigated for their roles in viral replication, expression and disease pattern as determined for HBV.

When GX-1, a highly pathogenic strain at 10^4^ TCID_50_ and three additional virus doses was inoculated in SPF chickens, 100% mortality was observed at 2 and 4 days post-inoculation (dpi) (Ren et al., 2019). After infection, the clinical signs of chickens were huddling in corners, ruffled feathers, depression, and decreased movement. The infected chicken livers were swollen and pale brown and contained necrotic foci. The pericardial sacs contained jelly-like fluid. Swollen and congested kidneys were also observed. In contrast to the nonpathogenic strain of FAdV-4, the pathogenic strain GX1 contains 43721 bp, 54.87% G+C content, and 38 open reading frames. This strain had 98.3% sequence identity with ON1.

## Roles and functions of structural proteins of FAdV-4 in viral pathogenesis

The complete genome sequence analysis of all FAdVs delineated that they share a common genome organization. The major structural proteins and some nonstructural proteins (Nsps) are located in the middle part of the genome.

### Hexon protein

Hexon, a monomer of FadV-4, is the most abundant structural protein, with 937 amino acid residues and a molecular weight of approximately 107 kDa. It is found in an abundance of 40 to 820 copies per virion and plays a vital role not only in genome organization but also in virus-neutralizing activity and serotype specificity (Roberts et al., 2006; Matsushima et al., 2011). Each hexon protein molecule consists of two conserved pedestal regions, P1 and P2, involved in hexon trimer formation and four loops (L1, L2, L3, and L4), with loops L1, L2 and L4 located on the external side of each hexon monomer; these loops are responsible for antibody binding, while L3 is situated internally and stabilizes the interface between P1 and P2. The loops containing several hypervariable regions (HVRs), in which four HVRs (HVR1-HVR4) are located in loop L1, two in loop L2 and one in loop L4, contain antigenic and immunogenic determinants and thus are used to differentiate 12 serotypes of FAdVs (Ganesh et al., 2001); however, in loop L3, there are no such determinants (Niczyporuk, 2018). The R188I mutation in the right side of HVR1 in L1 changed the conformation of the protein and thus was involved in the pathogenesis of FADV-4 (Wang et al., 2022a). Hypervariation in the amino acid sequence was reported in the HVRs. Due to its antigenic determinants, hexon has been widely used in molecular epidemiology studies. The L1 loop of the hexon protein is mainly used for serotyping, diagnosis and molecular typing of FAdV strains from different geographical locations worldwide (Raue and Hess, 1998; Marek et al., 2010; Pizzuto et al., 2010). It has been shown that the hexon protein physically binds the chaperone TCP-1-containing subunit (CCT7) and mediates viral replication. This finding highlights the potential of CCT7 to be an effective target protein for controlling FAdV-4 infection, as overexpression of CCT7 results in increased hexon expression, whereas knockdown of CCT7 results in decreased hexon protein expression (Gao et al., 2019).

The emergence of severe HHS in China due to a novel FAdV-4 strain compelled researchers to determine its virulence factor(s); therefore, in 2018, Zhang et al. investigated this issue (Zhang et al., 2021). Since the highly virulent isolates of FAdV-4 contain several mutations in the hexon gene, FAdV-4 infectious clones were generated by cloning the complete genome of CH/HNJZ/2015, a highly pathogenic isolate of FAdV-4, and ON1, a nonpathogenic strain, into the p15A-cm vector. A recombinant hexon-containing virus was constructed, and the pathogenesis of the rescued virus was compared with parent viruses, rON1and rHNJZ in SPF chickens aged 3 weeks. Chickens infected with rescued viruses harboring the hexon gene of HNJZ developed clinical signs similar to those of natural infection, indicating that the hexon protein is related to the pathogenesis of FAdV-4 (Zhang et al., 2021). Afterward, in 2021, Zhang et al. further investigated the specific amino acid residues in the hexon gene responsible for FAdV-4 pathogenesis. They generated a recombinant chimeric virus, the rHN20 strain, from a highly pathogenic strain by replacing the hexon protein of the nonpathogenic FAdV-4. After the inoculation of this chimeric strain into chickens, no mortality or even clinical signs appeared. These findings suggested that the hexon protein regulate the virulence of the novel FAdV-4 strain. Moreover, it was determined that with hexon, at position 188, the amino acid arginine (R) is important for determining the pathogenicity of this virus. The substitution of R with isoleucine (I) was performed to create the R188I mutant strain, and the rR188I strain and the wild-type strain replicate well *in vitro*; however, the rR188I strain is nonpathogenic according to serum neutralization *in vivo* and provides protection against HHS, suggesting that the R amino acid at position 181 is critical for pathogenesis (Zhang et al., 2021). After these findings were reported, Wang et al. in 2022 further delineated the function of the amino acid 188 in the hexon protein in both pathogenic and nonpathogenic FAdV-4 isolates (Wang et al., 2022a). A mutation in the hexon protein of HNJZ (a highly pathogenic FAdV-4 isolate) at amino acid residue 188 results in conformational changes; however, a similar kind of experiment with ON1 (a nonpathogenic FAdV-4 isolate) did not result in any conformational changes. Moreover, these results revealed that the role of amino acid 188 in the hexon protein relative to its virulence varies between different strains. In addition to the amino acid 188 in the hexon protein of FAdV-4, other factors must be present to induce severe HHS in chickens (Wang et al., 2022a).

### Fiber proteins

The first step in viral infection is the attachment of virus to host cell surface receptors and can have direct or indirect effects on pathogenesis, and these interactions are mediated by fiber proteins. This protein also plays a role in variations in virulence, virus neutralization, and cellular epitope binding (Pallister et al., 1996). Among the three capsid proteins of FAdV-4, fiber proteins protrude from one penton base of the viral particle and play an important role in viral infection and pathogenicity (Pan et al., 2017). The fiber patterns of the twelve FAdV serotypes showed an extreme degree of diversity. Unlike most serotypes of FAdV, serotypes 1, 4 and 10 have two distinct fiber proteins (fibers 1 and 2) on their surface (Marek et al., 2012). Both fiber proteins play important roles in viral pathogenesis (Wang W. et al., 2020). Notably, Fiber-1 of a novel hypervirulent FAdV-4 has 431 amino acids, whereas Fiber-2 has 479 amino acids (Liu et al., 2016), suggesting that Fiber-1 might be the short fiber of FAdV-4. Fiber-1 was shown to directly trigger FAdV-4 infection by binding its knob and shaft domains to domain 2 (the coxsackie and adenovirus receptor; CAR) of the host cell (Freimuth et al., 1999). In addition to being the foremost virulence determinant, the FAdV-4 Fiber-2 protein is a vaccine target that counters FAdV-4 infection upon vaccination. Numerous studies have shown that recombinant Fiber-2 can provide improved defense against a deadly encounter with FAdV-4 compared to that from extra capsid proteins, as indicated by the presence of Hexon, Penton and the all-important Fiber-1. Nonetheless, the Fiber-2 protein cannot induce the production of detectable neutralizing antibodies to counter FAdV-4 (Schachner et al., 2014). Although the Fiber1 complete protein or its knob domain trigger the production of neutralizing antibodies against FAdV-4, the efficiency of this protein for inducing the production of efficient antibodies still needs to be determined (Pan et al., 2020; Wang W. et al., 2020). The pathogenic potential of the fiber1 protein was evaluated in a study in which a fiber1 replacement mutant was constructed using the HNJZ strain. The rescued virus was tested in three-week-old SPF chickens, revealing that fiber 1 from ON1 mediated the induction of clinical signs similar to those of natural hypervirulent FAdV-4 infection, suggesting that the pathogenicity of FAdV-4 was independent of fiber 1 (Liu et al., 2020). Similarly, recombinant Fiber2-knob protein (F2-knob) was found to be immunogenic in 14-day-old SPF chickens (Song et al., 2023). The protective efficacy of F2-knob was evaluated in terms of virus shedding, clinical symptoms, mortality, and histopathology after FAdV-4 challenge. The antibody levels measured by ELISA were greater than those in chickens immunized with an inactivated vaccine against FAdV-4. Further experiments showed that F2-knob immunization significantly reduced viral shedding and provided complete protection against virulent FAdV-4, suggesting that F2-knob could be a vaccine candidate for controlling FAdV-4 (Song et al., 2023).

### Penton protein

Penton, a major structural protein, is responsible for the internalization of viruses during the infection cycle. Penton is composed of two units: a penton base and a projection (fiber) (Rashid et al., 2020). The function of penton in viral pathogenesis was evaluated by constructing a penton-replacement mutant using the HNJZ strain, and SPF chickens, aged three weeks were infected with the resultant strain. The results showed that the ON1 penton resulted in similar clinical signs as the natural hypervirulent FAdV-4, suggesting that the enhanced FAdV-4 virulence is independent of penton (Liu et al., 2020).

### Structural PX protein

The PX structural protein of FAdV-4 contains approximately 179 amino acid residues and has a molecular weight of 20 kDa; this protein also forms a complex with FAdV-4 genomic DNA. It has been reported that the PX proteins of several virulent FAdV-4 strains, such as HNJZ, SDDZ SXCZ, AHBZ, JSXZ and HuBWH in China are different from that of the nonpathogenic FAdV-4 ON1 strain by only two amino acids, at positions 11 and 129. The virulent strains had alanine substitutions (Liu et al., 2016) in place of threonine (T) at both positions (A11T, A129T), compared to the nonpathogenic FAdV4 ON1 strain. It has been found that amino acids at positions 11 and 129 (A11 and A129) are important for PX-induced apoptosis, as A11T and A129T mutations resulted in reduced apoptosis. Similarly, the R/K regions at positions 14 to 16 and 22 to 25 of PX are responsible transport the protein from cytoplasm to the nucleus. Thus, FAdV4-induced apoptosis mediates viral replication, which enhances FAdV4 infection (Liu et al., 2016; Zhao M. et al., 2020).

## Roles and functions of nonstructural proteins of FAdV-4 in viral pathogenesis

The nonstructural proteins (Nsps) of FAdV-4 play vital roles in its pathogenicity, genome replication and viral assembly. The 100k protein is 95 kDa, and contains approximately 798 amino acid residues. The 100K protein is encoded by the L4 gene during the last stage of infection and is involved in hexon trimerization and thus the efficient generation of progeny viruses (Koyuncu et al., 2013). The physical interaction between 100K and the host cellular protein HSC70 was verified through a coimmunoprecipitation (co-IP) assay in LMH cells, and this interaction is important for viral replication (Gao et al., 2022). This interaction between 100K of the virus and HSC70 of the host cell should be considered for the development of novel antiviral strategies against FAdV-4.

Maximum studies have focused on well-known proteins, however, studies about the roles and functions of unnamed ORFs is almost absent. Two ORFs i.e. ORF20A and ORF28, located in 3’ terminal region and ORF1B, located in the 5’ terminal region is unique to FAdV-4 and are absent in *Aviadenovirus*, and even in *Mastadenovirus.* The protein coding potential of ORF20A and ORF28 is extremely low and might act as regulatory genes that control the expression of other proteins (Gao et al., 2024). However, ORF1B could be only detected in the infected cells and not in mature virions. Moreover, the expression levels of ORF1B decreased gradually along FAdV-4 infection suggesting that this protein is an early gene transcript (Gao et al., 2024). The location of ORF1B is same as the E1A in *Mastadenovirus* members, indicating that this protein might be an early transcript which could counteract host immune responses and thus to regulate virus infection. Further investigations about this protein will dissect its role in the pathogenesis of this virus. The 33K Nsp play important role in capsid assembly and was found highly immunogenic (Morin and Boulanger, 1984). The 52/55K protein helps in host immune evasion by degrading PKR protein by a ubiquitin proteasome degradation system (Gao et al., 2024).

## The potential regulatory effect of FAdV-4 infection on ncRNAs

Several studies have indicated that viral infection induces modifications in the transcriptomes of target cells, including coding and noncoding RNAs (ncRNAs). MicroRNAs (miRNAs) are small ncRNAs approximately 20-24 nucleotides long. miRNAs either degrade or repress the translation of target mRNAs (Bartel, 2004). These miRNAs regulate various cellular processes i.e. differentiation (Kornfeld et al., 2021), apoptosis (Chen et al., 2021), autophagy (Li et al., 2020), development (Tsagakis et al., 2020), and tumorigenesis (Du et al., 2016), in addition to immune responses and virus entry (Xu et al., 2019). Host cellular miRNAs indirectly affect viral replication by targeting host cellular proteins involved in viral replication.

To dissect the roles of miRNAs in host, RNA sequencing was performed in LMH cells infected with FAdV-4 (Haiyilati et al., 2022). During this study it was found that the expression of 552 miRNAs in were altered. Twelve (12) miRNAs with altered expression levels were further selected for their roles in viral replication and apoptosis in host cells. Among these miRNAs, gga-miR-30c-5p was found to be involved in the inhibition of invasion and proliferation of glioma by targeting Bcl-2 (Yuan et al., 2021). Therefore, the role of gga-miR-30c-5p was further evaluated in response to FAdV-4 infection in host cells. The expression of gga-miR-30c-5p was significantly decreased during FAdV-4 infection. Furthermore, over expression of gga-miR-30c-5p mimics in LMH cells resulted in significant apoptosis, depicted by flow cytometry experiments whereas the reciprocal experiments delineated that apoptosis was inhibited by blocking the activity of gga-miR-30c-5p by using the inhibitors. These findings suggested that gga-miR-30c-5p act as pro-apoptotic in LMH cells that is down regulated during FAdV-4 infection (104).

In the same study (104), gga-miR-30c-5p was found to enhance FAdV-4 replication in LMH cells. The replication of FAdV-4 was assessed by the expression of Hexon and Px proteins. The expression of Hexon and PX proteins was significantly increased in FAdV-4 infected LMH cells with gga-miR-30c-5p transfection. In reciprocal experiments by inhibiting the activity of gga-miR-30c-5p by using the miRNA inhibitor, the replication of FAdV-4 was reduced (104). These findings suggested that gga-miR-30c-5p promote the replication of FAdV-4. Further to dig out the mechanism, Mcl-1 was identified as direct target of gga-miR-30c-5p, as knock down of this protein increased apoptosis in LMH cells, whereas over expression of Mcl-1 counteracted gga-miR-30c-5p or FAdV-4 induced apoptosis. Therefore, gga-miR-30c-5p promote FAdV-4 induced apoptosis by targeting Mcl-1 directly and facilitate FAdV-4 replication inside host cells (104).

In another study miRNA-seq analysis was performed in LMH cells in order to understand the entry of FAdV-4 into host cells (Wu et al., 2020). During this study the expression of 785 miRNAs was altered, and gga-miR-15c-3p, gga-miR-148a-3p, and gga-miR-148a-5p were identified for the first time to be associated with FAdV-4 infection (Wu et al., 2020). Furthermore, the expression of gga-miR-128-2-5p was also altered and luciferase assay showed that OBSL1 was its true target. Overexpression of gga-miR-128-2-5p resulted in the down regulation of OBSL1 and inhibited the entry of FAdV-4 into the cells. These findings suggested that gga-miR-128-2-5p play a crucial role in the entry of FAdV-4 into host cells by targeting OBSL1 (Wu et al., 2020).

In another study the role of gga-miR-181a-5p in regulating FAdV-4 replication was elucidated (Yin et al., 2021). During FAdV-4 infection the expression of gga-miR-181a-5p was induced in LMH cells and was found to promote FAdV-4 replication. The stimulator of interferon genes (STING) was found to be the direct target of gga-miR-181a-5p, and knockout of STING increased FAdV-4 replication, whereas its over expression decreased FAdV-4 replication. These findings revealed that gga-miR-181a-5p promote FAdV-4 replication (Yin et al., 2021).

Long noncoding RNAs (lncRNAs) are also a class of ncRNAs, are greater than 200 nucleotides in length. Since lncRNAs do not contain an open reading frame, they cannot encode functional proteins (Kapranov et al., 2007; Cao, 2014). Like miRNAs, lncRNAs affect the transcription and translation of both host and virus genes, the stability of mRNAs and the host antiviral response (Liu and Ding, 2017). It has been reported that viral infection alters the lncRNA transcription profile of infected host cells, which is involved in the establishment and maintenance of persistent infection (Meng et al., 2017). It has been reported that FAdV-4 induces apoptosis in LMH cells by modulating the transcription of the BMP4 gene, which is targeted by lncRNA 54128 (Wen et al., 2021). The miRNAs and lncRNAs whose transcription is induced during FAdV-4 infection, as well as their target mRNAs, are listed in **Table 1**.

**Table 1:** miRNAs, lncRNAs and their targets during FAdV-4 infection.

S. No	ncRNA	Target protein(s)	Protein function	Reference
1	gga-miR-7475-5p	CREBZF/LFNG	Host cell response viral infection	(Smith et al., 2010; Wu et al., 2020)
2	gga-miR-128-2-5p	OBSL1	FAdV-4 entry to the host cell	(Wu et al., 2020)
3	novel-miR271	EGR1	Virus infection	(Wu et al., 2020; Wang XP. et al., 2022)
4	gga-miR-30c-5p	Mcl-1	Viral replication	(Haiyilati et al., 2022)
5	gga-miR-205c-3p	FRMD6	Apoptosis	(Angus et al., 2012; Wu et al., 2020)
6	miR-181a-5p	STING	Viral replication	(Kapranov et al., 2007)
7	gga-miR-12223-3p	SERTAD2	Carcinogenesis	(Wu et al., 2020; Chen et al., 2022)
8	gga-miR-6654-3p	NFATC1	T-cell regulation	(Giri et al., 2022)
9	gga-miR-12245-3p	AQP11	Transport function	(Gorelick et al., 2006)
10	miR-27	SNAP25/TXN2	Virus entry	(Machitani et al., 2017)
11	ENSGALG00000055015	BMP4	Apoptosis	(Liu and Ding, 2017; Cao et al., 2020)
12	ENSGALG00000054172	SOCS2	Immune-related cancer	(Liu and Ding, 2017; Sobah et al., 2021)
13	XLOC_026155	FOXO3	Apoptosis	(Lim et al., 2021)
14	ENSGALG00000050472	FOXO3	Apoptosis	(Lim et al., 2021)
15	ENSGALG00000048532	SLC40A1	Iron metabolism	(Wang et al., 2023)
16	XLOC_003734	MKP3	Apoptosis	(Rössig et al., 2000; Liu and Ding, 2017)

## The role of FAdV-4 infection in host immune responses

Although limited information is available regarding FAdV infection and the host immune response, it is broadly believed that certain components of innate immunity are crucial for combatting FAdV infection in fowl. One such innate immune component is avian ß-defensins (AvBDs), which play a pivotal role in viral infections. It has been reported that most AvBDs (AvBD 5, 7, 8, 9, and 13) were upregulated in specific FAdV-4-infected fowl tissues, and a positive correlation was observed between the FAdV-4 genome levels and upregulation of the aforementioned AvBDs, suggesting the important role of AvBDs in the immune response to FAdV-4 infection (Haiyilati et al., 2021). In addition, the host immune system initiates an immune response by inducing the production of antiviral factors after recognizing viral pathogen-associated molecular patterns through pattern recognition receptors (PRRs). Consequently, several immunity-related pathways, including but not limited to the Toll-like receptor (TLR) signaling pathway, MyD88-mediated FAdV-4-induced inflammation and the cytokine−cytokine receptor interaction pathway, are activated after FAdV-4 infection (Li et al., 2018; Zhao W. et al., 2020). The cyclic GMP-AMP (cGAMP) synthase (cGAS) is a DNA sensor that trigger the induction of innate immune responses to produce IFN-I and proinflammatory cytokines (Wang J. et al., 2020).The cGAS has been well studied in mammals however, in the chickens the Cgas (chcGAS) has not been thoroughly studied. The cGAS recognizes pathogens that contain DNA whereas, cells lacking cGAS do not respond efficiently to DNA viruses (Lam et al., 2014; Ma et al., 2015). Soon after binding the double stranded DNA inside cytosol, the cGAS converts guanosine 5’ –triphosphate and adenosine 5’ –triphosphate into 2’,3’ –cyclic GMP-AMP (2’, 3’-cGAMP) that serve to trigger the stimulator of IFN genes (STING) (Li et al., 2013; Sun et al., 2013). STING undergoes significant conformational changes when bind to 2’,3’ –Cgamp, recruit TANK binding kinase (TBK1), leading to the phosphorylation of IFN-regulatory factor 3 (IRF3) and nuclear factor-κB (NF-κB), and finally expresses IFNs and proinflamatory cytokines (Tanaka and Chen, 2012; Ablasser et al., 2013). cGAS can also translocate into the nucleus and suppresses homologous-recombination-mediated DNA repair in response to DNA damage (Liu et al., 2018). The role of chcGAS was well evaluated by Wang et al. who cloned the complete open reading frame of chcGAS from cDNA derived from ileum (Wang J. et al., 2020). The phylogenetic analysis and multiple sequence alignment delineated that chcGAS was homologous to cGAS of mammals. The chcGAS was found to be highly expressed in ileum and bone marrow. Moreover, chcGAS was highly expressed inside cytoplasm and partially co-localized with endoplasmic reticulum (ER). Over expression of chcGAS resulted in increased transcription of proinflammatory cytokine i.e. interleukin 1β (IL-1β) and IFN-β in a dose dependent manner in CEF and LMH cells. Moreover, transfection of herring sperm DNA (HS-DNA) and poly (dA:dT) but not poly (I:C) further enhanced chcGAS-induced IL-1β and IFN-β expression, indicating that chcGAS respond only to dsDNA in LMH and CEF cells. Furthermore, chcGAS-induced IL-1β and IFN-β activation was found to be dependent on STING signaling pathway. Finally, it was shown that chcGAS was involved in sensing FAdV-4 in LMH cells, and knock down of chcGAS promoted FAdV-4 infection in LMH cells. All this indicate that chcGAS play an antiviral role during FAdV-4 infection through STING signaling pathway to induce the expression of IL-1β and IFN-β (Wang J. et al., 2020). Cytokines are proteins or glycoproteins that are secreted by immune cells after viral infections. Usually, the pro-inflammatory cytokines and IFN-I are expressed quickly to counteract viral infections. The infection of chickens with FAdV-9 increased the expression levels of IFN-α, IFN-γ, and IL-12 in bursa of Fabricius, spleen, and liver (Deng et al., 2013). The infection of chickens with FAdV-8 increased the expression levels of IFN-γ mRNA and decreased IL-10 levels in the spleen (Grgić et al., 2013b). Similarly, the infection of FAdV-4 has resulted in the increased expression levels of IL-1β, IL-2, IL-6, IL-8, IL-10, IL-18, IFNγ, and TNF-α in the *in vivo* and *in vitro* conditions (Grgić et al., 2013a; Niu et al., 2019; Zhao W. et al., 2020).

In another study, the differences in the innate immune responses both *in vivo* and *in vitro*, between chickens and ducks were delineated by using SD0828 strain of FAdV-4 (Li et al., 2018). The mRNA levels of pro-inflammatory cytokines i.e. IL-6 and IL-8 and interferon stimulated genes (ISGs) such as, Mx and OAS were significantly increased in chicken embryo fibroblasts (CEFs) and duck embryo fibroblasts (DEFs). The significantly highly expression of IL-6 and IL-8 also produced severe inflammatory responses in the infected tissues, resulting in deaths at higher rate. Although the mRNA levels of ISGs were also increased significantly, they did not withstand the inflammatory storm in the infected organisms (Li et al., 2018). Another study performed by He et al. delineated that mRNA levels of IFNs were increased from 24h to 72h post FAdV-4 infection in LMH cells and livers however, the FAdV-4 viral load still increased significantly (He et al., 2023). These findings suggested that FAdV-4 escaped the host innate immune responses. Moreover, in the same study it was revealed that only the protein levels and not the mRNA levels of Protein Kinase R (PKR) were degraded after 48 h of FAdV-4 infection inside the host cells. Furthermore, the 52/55 K protein was ubiquitinylated; leading to the degradation of protein kinase R (PKR) inside host cells through the proteasomal degradation pathway. These results revealed that the 52/55 K protein of FAdV-4 degrades PKR through the ubiquitin−proteasome pathway and helps FAdV-4 evade host immune responses (He et al., 2023).

Cytokines contribute to both adaptive and innate immunity to viral infection and have therefore both local and systemic effects. Higher immune responses sometimes produce negative effects to the host, for example, malaise, fever, and inflammatory damage are observed when increased levels of cytokines are produced (Wang et al., 2022b). High inflammatory damage has been observed in FAdV-4 infected chickens, and a few studies have elaborated the cytokine secretion. High expression of cytokines is observed in the organs targeted by the virus, however, function of each of each cytokine is still to be determined. Interleukins (ILs), Interferons (IFs), transforming growth factors (TGFs), TNGs, chemokines and colony stimulating factors (CSFs) all are inflammatory cytokines. In case of FAdV-4, the well-studied cytokines are; Tumor necrosis factors (TNFs), IFNs, and ILs. Usually cytokines affect the production of their own as well other cytokines production to make a complex cytokine network. The effect of cytokines mostly depends on timing, duration and the target organ, however, is still difficult to make a final conclusion whether a cytokine protects or contribute to hosts damage caused by FAdV-4 infection. When the MDA5, NLRP3, and TLRs recognize the FAdV-4, cytokines expression is triggered by translocation of NF-κB from cytoplasm to nucleus. The TNFs and IL-1β trigger the activation of NLRP3 inflamasomes, which in turn enhance the production of proinflammatory cytokines. FAdV-4 is known to trigger both pro and anti-inflammatory cytokines in the main targeted organs. The production of proinflammatory cytokines has been found to cause organ failure and even death of the host (Chen et al., 2018; Nile et al., 2020).

Pericardial effusion is also observed in chickens infected by FAdV-4, which could lead to a conclusion that excessive inflammatory response may result in organ exhaustion and finally to death. It has been observed experimentally that chicken infected with pathogenic FAdV-4 strain, CH/HNJZ/2015 showed that expression levels of mRNA encoding pro inflammatory cytokines i.e. IL-8 and IL-18 and antiviral cytokine, IFN-β were significantly higher compared to the chickens infected with non-pathogenic FAdV-4 strain ON1 in various organs (Grgić et al., 2013a). The role of cytokines in host immune responses is important in determining their action as friend or enemy. Previously IL-1β, IL-2, IL-4, IL-6, and IL-18 have showed potential treatment or vaccine adjuvants for avian influenza viruses (AIV), Newcastle disease, and infectious bursal disease virus (IBDV), therefore, the possible therapeutic application of these cytokines against FAdV-4 infection warrants future investigations (Wang et al., 2022b).

Furthermore, infection with AD234, a virulent FAdV-4 strain, decreased the number of CD3-, CD4-, and CD8-positive T cells in the spleen, as well as the number of CD4- and CD8-positive T lymphocytes in the thymus. Lymphocyte numbers were also decreased in the bursa of Fabricius. These observations suggested that FAdV-4 escapes the adaptive immune response of host chickens (Haiyilati et al., 2021). Infection of SPF chickens with FAdV-4 resulted in atrophy of the bursa of Fabricius, a decrease in lymphocyte count and a decrease in the number of B and T cells in lymphoid organs (El-Shall et al., 2022). The PX protein is critical for apoptosis, and lymphocyte apoptosis causes immunosuppression. However, the detailed mechanism by which FAdV-4 induces apoptosis leading to immunosuppression still needs to be investigated (Haiyilati et al., 2021). Species immunity and genetic background might also be factors involved in immune evasion by FAdV-4.

## Conclusions

FAdV-4 has spread globally, is highly pathogenic and has caused substantial economic loss in the associated industry. FAdV-4 proteins, especially structural proteins, play important roles in pathogenesis; however, mutations in these proteins have been identified through continuous surveillance, and these mutations may cause the development of even more hypervirulent strains of FAdV-4. Therefore, continuous screening for new mutations in viral proteins needs to be performed to help in the design of effective vaccines against FAdV-4. In recent years, ncRNAs have been used for therapeutic purposes to treat different diseases; therefore, the therapeutic potential of ncRNAs that are regulated after FAdV-4 infection must be further explored. Regarding the immune responses, identification of specific genes of FAdV-4 regulating cytokines production will help in the robust understanding FAdV-4 infection.

## Author contributions

FR: Writing – original draft, Writing – review & editing. ZxX: Conceptualization, Funding acquisition, Investigation, Resources, Supervision, Writing – review & editing. YW: Investigation, Writing – review & editing. ZqX: Writing – review & editing. LX: Writing – review & editing. ML: Investigation, Writing – review & editing. SL: Investigation, Writing – review & editing.
